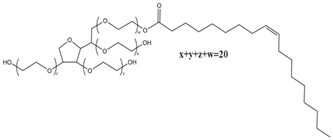# Systematic Investigation of the Effect of Non-Ionic Emulsifiers on Skin by Confocal Raman Spectroscopy—A Comprehensive Lipid Analysis

**DOI:** 10.3390/pharmaceutics12030223

**Published:** 2020-03-02

**Authors:** Yali Liu, Dominique Jasmin Lunter

**Affiliations:** Department of Pharmaceutical Technology, Eberhard Karls University, Auf der Morgenstelle 8, 72076 Tuebingen, Germany; cpuyali@gmail.com

**Keywords:** non-ionic emulsifiers, intercellular lipids, confocal Raman spectroscopy (CRS), polyethylene glycol alkyl ethers, polyethylene glycol sorbitan fatty acid esters

## Abstract

Non-ionic emulsifiers are commonly found in existing pharmaceutical and cosmetic formulations and have been widely employed to enhance the penetration and permeation of active ingredients into the skin. With the potential of disrupting skin barrier function and increasing fluidity of stratum corneum (SC) lipids, we herein examined the effects of two kinds of non-ionic emulsifiers on intercellular lipids of skin, using confocal Raman spectroscopy (CRS) with lipid signals on skin CRS spectrum. Non-ionic emulsifiers of polyethylene glycol alkyl ethers and sorbitan fatty acid esters were studied to obtain a deep understanding of the mechanism between non-ionic emulsifiers and SC lipids. Emulsifier solutions and dispersions were prepared and applied onto excised porcine skin. Water and sodium laureth sulfate solution (SLS) served as controls. SC lipid signals were analysed by CRS regarding lipid content, conformation and lateral packing order. Polyethylene glycol (PEG) sorbitan esters revealed no alteration of intercellular lipid properties while PEG-20 ethers appeared to have the most significant effects on reducing lipid content and interrupting lipid organization. In general, the polyoxyethylene chain and alkyl chain of PEG derivative emulsifiers might indicate their ability of interaction with SC components. HLB values remained critical for complete explanation of emulsifier effects on skin lipids. With this study, it is possible to characterize the molecular effects of non-ionic emulsifiers on skin lipids and further deepen the understanding of enhancing substance penetration with reduced skin barrier properties and increased lipid fluidity.

## 1. Introduction

Skin represents the largest organ of the human organism and forms the outermost barrier film that protects the human body from external environment impacts and exogenous irritations and corrosions. Stratum corneum (SC) is the uppermost layer of the skin, composed of a highly ordered, multilamellar lipid matrix with embedded flattened, keratin-filled corneocytes [1,2]. The particular intercellular lipids in SC consist of ceramides, free fatty acids and cholesterol in an approximately equimolar ratio [3,4]. It has been well accepted that intercellular lipids in SC play an important role in maintaining skin barrier function and keeping the skin in a proper hydration state [5]. The removal and organizational alteration of intercellular lipids would disrupt the barrier function from multiple aspects and deteriorate into some chronic skin diseases [6,7]. With the importance of assuring the solid structures and ordered properties of skin lipids, it is essential to monitor the molecular lipid interactions with exposed substances and maintain the protective barrier state for further optimization of dermatological treatments.

In everyday life, the skin is exposed to different environments and mostly exposed to various sanitary and cosmetic products and cleansers. Among the main components of them, non-ionic emulsifiers have been widely used and become the potential class to be exposed on the skin and may simultaneously interact with molecular skin components [8,9]. Although non-ionic emulsifiers are usually considered to be relatively safe and better tolerated in comparison to cationic and anionic emulsifiers, they have been proved having the potential to interact with biological membranes, especially skin, with their increasing applications [10,11,12]. In particular, recent studies from our group found that polyethylene glycol (PEG)-20 glycerol monostearate displayed negative effects on skin lipid extraction and structural disruption while polysorbate 80 reflected no such effects [13,14]. For this reason, the selection and usage of non-ionic emulsifiers in pharmaceutical and cosmetic formulations are currently of high interest. In order to gain mechanistic insights into their skin effects, PEG alkyl ethers and sorbitan fatty acid esters (e.g., polysorbate or Tween) were taken into consideration which are both non-ionic PEG derivatives. They are composed of a hydrophilic polyoxyethylene head group and a lipophilic alkyl chain. Keeping constant either of these two parts, the other part of size and chain length etc. would be potential parameters to monitor the governing rules [15]. Furthermore, these two groups of emulsifiers are widely applied in cosmetic and dermal products due to their solubility and viscosity properties with low toxicity according to the existing safety assessments identified in previous studies [16,17]. We therefore chose to further investigate these two groups of emulsifiers in a systematic approach and evaluate their possibilities for prospective development of skin products with relatively mild effects on skin lipids [18].

Up to now, considerable number of studies have made efforts on obtaining more information about the quantitative and structural properties of skin lipids [19,20,21]. The means of small- and wide-angle X-ray scattering, neutron scattering, and liquid chromatography coupled with mass spectrometry have been intensively applied. However, those methodologies have potential risks of sample contamination, being time-consuming and sample destruction [22,23]. In this area confocal Raman spectroscopy (CRS) has emerged as a promising non-invasive tool in skin research and caught increasing attention for skin characterizations such as skin penetration and permeation of topically applied materials [24,25]. CRS is also an efficient and label-free method and has been increasingly used to study the macroscopic alterations of skin properties with humidity changes, age difference and hair removal comparisons [26,27,28]. Detailed information about biochemical molecules can be obtained from CRS, including the chemical structure, phase and molecular interactions. Recent findings from our group have demonstrated that CRS could be used as an alternative method to analyze lipid extraction and conformation in SC [13,14]. Based on this proof and listed advantages, CRS was used in this study as a useful and convenient approach for lipid analysis.

For studying the physiological parameters of SC lipids, many researchers have focused on finding spectral signals in CRS for skin research [29,30]. Those spectral signals in this study are the identified Raman bands associated with molecular vibrations of lipids inside the skin. They are originated from the methylene (−CH_2_−) and methyl (−CH_3_) groups in lipid molecular structures. Using them a comprehensive study measuring lipid content, conformational order, lateral packing order and SC thickness could be conducted at the same time. However, the skin CRS spectrum is extremely complex and the lipid-derived Raman bands usually overlap with bands originating from molecular vibrations of proteins [19,31]. In order to track the individual lipid-related spectral signals, a Gaussian-function based mathematical procedure could be applied on account of a previous report from Choe et al. [32].

As we know, many hypotheses regarding the interactions between emulsifiers and skin are still not well grounded [33], making it essential to perform a systematic investigation of non-ionic emulsifiers. Therefore, the aim of this study is to use different lipid-related spectral signals to analyse the interactions between non-ionic emulsifiers and intercellular lipids. PEG alkyl ethers and sorbitan fatty acid esters were selected. To the best of our knowledge, this is the first systematic report considering PEG derivatives with different number of oxyethylene groups and different hydrophobic alkyl chain lengths using CRS to better understand their mechanism of influence on skin components. Results should be helpful to find a rule for further selections of non-ionic emulsifiers which is beneficial to the development of skin products.

## 2. Materials and Methods 

### 2.1. Materials

PEG alkyl ethers including PEG-2 oleyl ether (O2), PEG-10 oleyl ether (O10), PEG-20 oleyl ether (O20), PEG-2 stearyl ether (S2), PEG-10 stearyl ether (S10), PEG-20 stearyl ether (S20), PEG-2 cetyl ether (C2), PEG-10 cetyl ether (C10), PEG-20 cetyl ether (C20) were purchased from Croda GmbH, (Nettetal, Germany). PEG sorbitan fatty acid esters containing PEG-20 sorbitan monopalmitate (Polysorbate 40, PS40), PEG-20 sorbitan monostearate (Polysorbate 60, PS60), PEG-20 sorbitan monooleate (Polysorbate 80, PS80) were obtained from Caesar & Loretz GmbH (Hilden, Germany). Sodium lauryl sulfate (SLS) was obtained from Cognis GmbH & Co. KG (Düsseldorf, Germany). Trypsin type II- S (lyophilized powder) and trypsin inhibitor (lyophilized powder) were obtained from Sigma-Aldrich Chemie GmbH (Steinheim, Germany). Parafilm^®^ was from Bemis Company Inc., (Oshkosh, WI, USA). Sodium chloride, disodium hydrogen phosphate, potassium dihydrogen phosphate, and potassium chloride were of European Pharmacopoeia grade. All aqueous solutions were prepared with ultra-pure water (Elga Maxima, High Wycombe, UK). Porcine ear skins (German land race; age: 15 to 30 weeks; weight: 40 to 65 kg) were provided by Department of Experimental Medicine at the University of Tuebingen. The Department of Pharmaceutical Technology at the University of Tuebingen has been registered for the use of animal products [13]. 

### 2.2. Preparation of Dermatomed Porcine Ear Skin

Porcine ear skin was selected as substitute for human skin in this study due to their histologically and morphologically similarity with human skin [34,35]. Porcine ears used in this study were achieved from the Department of Experimental Medicine of the University Hospital Tuebingen. The live animals used were kept at the Department of Experimental Medicine and sacrificed in the course of their experiments, which are approved by the ethics committee of the University Hospital Tuebingen. Those ears were received directly after the death of the animals. Prior to study start, the Department of Pharmaceutical Technology has registered for the use of animal products at the District Office of Tuebingen (registration number: DE 08 416 1052 21). Fresh porcine ears were cleaned with isotonic saline. Full-thickness skin was removed from cartilage and gently cleaned from blood with cotton swabs and isotonic saline. The obtained postauricular skin sheets were then dried with soft tissue, wrapped with aluminium foil and stored in freezer at −30 °C. On the day of experiment, skin sheet was thawed to room temperature, cut into strips of approximately 3 cm width and stretched onto a Styrofoam plate (wrapped with aluminium foil) with pins to minimize the effect of furrows. Skin hairs were trimmed to approximately 0.5 mm with electric hair clippers (QC5115/15, Philips, Netherlands). Subsequently, the skin was dermatomed to a thickness of 0.8 mm (Dermatom GA 630, Aesculap AG & Co. KG, Tuttlingen, Germany) and punched out for circles to a diameter of 25 mm.

### 2.3. Incubation of Porcine Ear Skin in Franz Diffusion Cells

Franz diffusion cells have been commonly used as a specific analytical setup for ex vivo determination of skin absorption. Here, degassed, prewarmed (32 °C) phosphate buffered saline (PBS) was used as receptor fluid and filled in the Franz diffusion cells of 12 mL. The stirring speed of the receptor fluid was 500 rpm. The dermatomed skin circles were mounted onto the cells with donor compartment on above. The equipped Franz diffusion cells were put into water bath with temperature of 32 °C. After a short equilibrium of 30 min, 1 mL of each emulsifier solution/dispersion was applied to each skin sample (all non-ionic emulsifiers used in this study are listed in Table 1 with detailed information). Then, a piece of parafilm was capped onto each donor compartment to prevent evaporation. After 4 h incubation, skin samples were removed from cells and each skin surface was gently washed and cleaned with isotonic saline and cotton swabs for 30 times in order to remove the remaining samples and avoid erroneous measuring result. Finally, the actual application area (15 mm in diameter) was punched out and patted dry with cotton swabs. This part of the method has been detailly described by our group [13].

### 2.4. Isolation of Stratum Corneum

The stratum corneum was isolated following the trypsin digestion process as described by Kligman et al. and Zhang [14,36]. This isolation procedure has been proved to have no influence on the lamellar lipid organization [37]. The obtained skin circles (with diameter of 15 mm) from last step were placed dermal side down on filter paper soaked with a 0.2% trypsin and PBS solution. After the incubation of skin sample for overnight at room temperature, digested SC was peeled off gently and immersed into 0.05% trypsin inhibitor solution for 1 min. Afterwards, the isolated SC was washed with fresh purified water for min. five times to remove the underlayer tissues. The final obtained SC sheet was then placed onto glass slide and stored in desiccator to dry for min. three days.

### 2.5. Confocal Raman Spectroscopy (CRS)

In order to investigate the effects of different non-ionic emulsifiers on SC, CRS served as the primary instrument to detect their differences. After drying, the SC sheets were taken out of the desiccator and fitted onto the scan table of alpha 500 R confocal Raman microscope (WITec GmbH, Ulm, Germany). This CRS device was equipped with a 532-nm excitation laser, UHTS 300 spectrometer and DV401-BV CCD camera. To avoid the damage of skin sample due to higher laser intensity, the laser power used was 10 mW, which could be adjusted using the optimal power meter (PM100D, Thorlabs GmbH, Dachau, Germany). A 100× objective with numerical aperture of 0.9 (EC Epiplan-neofluor, Carl Zeiss, Jena, Germany) was used to focus the light on skin surface. The backscattered light from the skin was then dispersed by an optical grating (600 g/mm) to achieve the spectral range from 400–3800 cm^−1^. Collected scattered light was analysed on a charge-coupled device (DV401-BV CCD detector) which had been cooled down to −60 °C in advance. The CRS measurements were performed based on a method developed by Zhang et al. [13,14].

### 2.6. Determination of Skin Surface and Thickness

In order to achieve spectral signals of lipids from skin surface and measure SC thickness at the same time, the spectra were detected with the focus point moving from −50 μm beneath the skin to 50 μm above the skin. The spectra were recorded with the step size of 1 μm. The skin surface was determined using the intensity difference of keratin signal (ν (CH_3_), 2920–2960 cm^−1^). The area under the curve (AUC) of the keratin peak was calculated and plotted against depth. While the intensity of the keratin signal reaches the half maximum, the laser spot would be located at the boundary between glass slide and skin bottom or the boundary between skin surface and air [13,38]. So that the spectrum extracted from the boundary between skin surface and air was regarded as skin surface and used for lipid signal analysis. Moreover, with the description above, the full width of half maximum (FWHM) could serve as the thickness of skin sample. 

### 2.7. Lipid Signals in Fingerprint Region

#### 2.7.1. C–C Skeleton Vibration Mode

**Figure 1:**
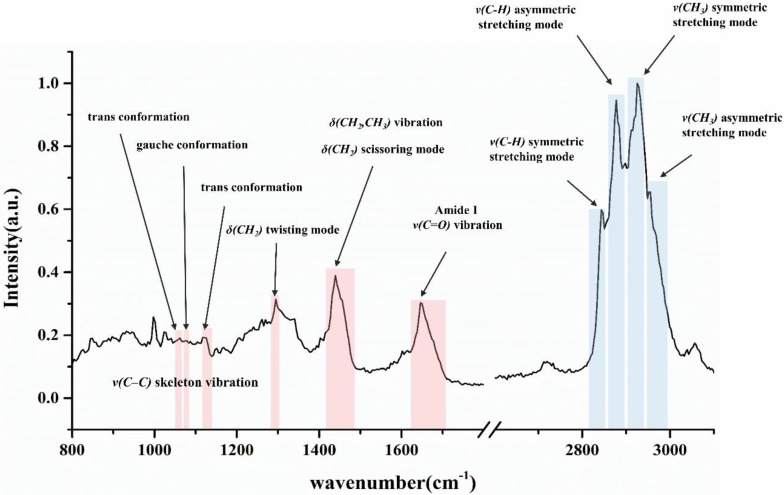
Major band assignments of CRS spectrum obtained from skin sample. The red/blue areas represent the specific peak referring to different molecular vibrations. The break on the axis of wavenumber separates the fingerprint region (left side) and high wavenumber region (right side). The peaks assigned to trans and gauche conformations are both originated from C–C skeleton vibration.

The first three small peaks in Figure 1 highlighted in red are assigned to the vibration of C–C skeleton. They are sensitive to the trans–gauche conformational order of long chain hydrocarbons which exist mostly in intercellular lipids [39,40]. The peaks located at 1060 cm^−1^ and 1130 cm^−1^ arise from all-trans conformation which stand for a more ordered state of lipids. The peak at 1080 cm^-1^ corresponds to the gauche conformation which represents a more disordered state of lipids [41,42]. In this case, PCA analysis and polynomial background subtraction are needed to remove the noise and obtain a more precise result. On the other hand, the band at 1130 cm^−1^ contains part of the contribution of keratin at 1125 cm^−1^. As a result, an adequate integration area is selected to eliminate the influence of keratin peak. Then, the conformational order could be calculated with the ratio of AUC of those three peaks: conformational order = AUC_1080_ / (AUC_1060_ + AUC_1130_) as originally described by Snyder, et al. [43]. Thus, a higher value of conformational order represents an indication to the gauche conformation and disordered state of lipids.

#### 2.7.2. CH_2_ Twisting and Scissoring Mode

The peak located at about 1300 cm^−1^ in Figure 1 is assigned to the CH_2_ twisting mode. The shift of this peak could also indicate the order or disorder of lipid conformations because of the sensitivity of this peak to hydrocarbon chains [44]. The broadening and shift to a higher wavenumber of this peak indicates a tendency of intercellular lipids turning into a more disordered and gauche conformation state.

Apart from the detection of conformational order on Raman spectra, vibrational characteristics are also convenient to determine the lateral packing state (orthorhombic order, hexagonal order, and liquid-like chain packing) influenced by the intramolecular interactions [44]. The CH_2_ scissoring band at 1430–1470 cm^−1^ reflects the nature of lateral packing between ceramide molecules. The shift of this peak to a higher wavenumber in the Raman spectrum stands for a more hexagonal or even liquid like packing state.

#### 2.7.3. CH_2_ and CH_3_ Stretching and C=O Vibration Mode

The last two bands marked in fingerprint region of Figure 1 are assigned to δ (CH_2_, CH_3_)– mode at 1425–1490 cm^−1^ and ν (C=O)– mode at 1630–1710 cm^−1^ respectively. The band at 1630–1710 cm^−1^ arises from the Amide I mode which showed the least variation within one donor or among different donors. Thus, this band has been often used in the normalization of other Raman peaks derived from SC [30,42]. The band at 1425–1490 cm^−1^ originates from both keratins and lipids. In this study, this band was regarded as “lipids-peak” for calculation of lipid content. 

Based on the equation of Normalized lipids = AUC_1425–1490_/AUC_1630–1710_, the lipid-keratin peak was normalized by the amide I peak. The final calculated result would indicate the content variation of lipids. Therefore, the lipid content in fingerprint region in this study was calculated as equation above.

### 2.8. Lipid Signals in High Wavenumber Region

In order to achieve further information about lipid content and lateral packing order state for a more precise and forceful result, the lipid-keratin peak (2800–3030 cm^−1^) in the HWN region was taken into consideration. This band is also derived from the CH_2_ and CH_3_ vibrations.

#### 2.8.1. Gaussian Deconvolution Process

As depicted in Figure 1, it is obvious that the peaks originated from lipids and keratins overlapped and formed a peak with higher intensity and broad width. In order to track the information of each peak individually, a mathematical process based on Gaussian functions was employed. As described by Choe, the Gaussian function type exhibited the least fitting error and demonstrated to be the best fitting function for Raman spectra of the skin [32]. 

**Figure 2:**
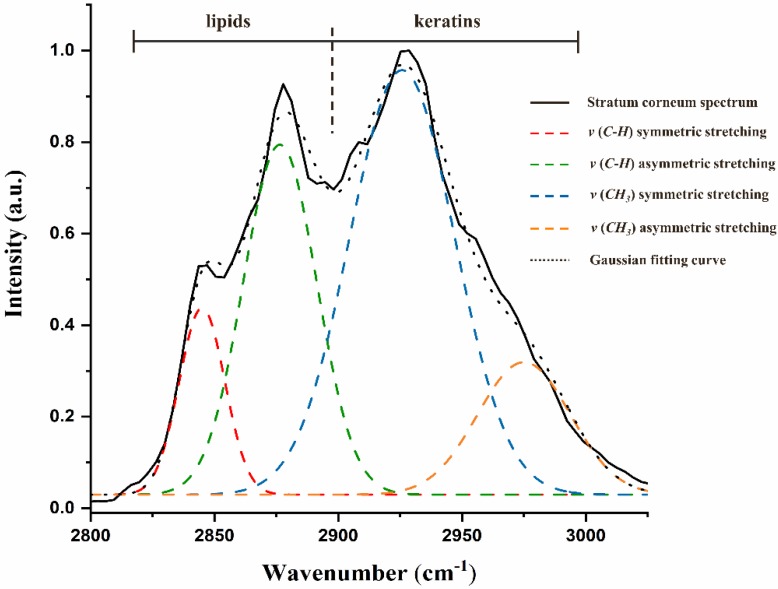
Deconvolution of lipid-keratin peak from skin spectrum by using four Gaussian peaks in high wavenumber region. The assignments were labelled with different colours.

In this study, the obtained spectrum of SC was deconvoluted into four Gaussian peaks automatically with the application of curve fitting toolbox on Matlab software (version R2019a, MathWorks GmbH, Natick, MA, USA). The summation of four Gaussian functions was used as the fitting formula. In order to achieve reproducible result and reduce the fitting error, the Gaussian peak maximum positions and FWHMs were allowed to vary in defined intervals only. Afterwards, a non-linear iteration process was applied. It can be seen from Figure 2 that each fitted peak was labelled in different colour. The goodness of fitting results could also be generated automatically with all R^2^ above than 0.98.

#### 2.8.2. ν (C–H) Symmetric and Asymmetric Stretching

As illustrated in Figure 2, peaks located at 2850 cm^−1^ and 2880 cm^−1^ stand for the ν (C–H) symmetric and ν (C–H) asymmetric stretching mode respectively which are both derived from the vibration of lipids. These two peaks are sensitive to the packing order of alkyl chains of lipids. Based on some research, the ratio of the intensities of these two peaks could be used as a sign of crystalline phase of intercellular lipids [39,45]. In this study, peak areas after deconvolution were applied for the calculation of lateral packings Ratio_lat_ = AUC_2880_/AUC_2850_ according to ref. [46]. This procedure has effectively eliminated the influence of multi-peak overlap phenomenon. Hereby, the higher Ratio_lat_ represents a prevalence towards highly crystalline and orthorhombic phase. The lower Ratio_lat_ reveals a tendency towards disordered and liquid-like phase.

#### 2.8.3. ν (CH_3_) Symmetric and Asymmetric Stretching

As is shown in Figure 2, the blue and orange peaks at 2930 cm^−1^ and 2980 cm^−1^ arise from the contribution of keratins and originated from the ν (CH_3_) symmetric and ν (CH_3_) asymmetric stretching separately. Previous studies often use the area of these two keratin related peaks to normalize lipid related peaks in order to determine the lipid content [47,48]. As described in this study, the AUC extracted directly in high wavenumber would contain the contribution of adjacent peaks. The deconvolution process would exclude the influence of peak superposition. As a result, the Gaussian areas after deconvolution were employed for calculation of lipid content: Normalized lipid = (AUC_2880_ + AUC_2850_)/(AUC_2930_ + AUC_2980_).

### 2.9. Data Analysis

#### 2.9.1. Raman Spectra Pre-Processing

The initial processing step of Raman spectra usually included the spectral cosmic ray removal, smoothing as well as background subtraction which were all performed by the WITec Project Software (WITec GmbH). Referring to the smoothing process, Savitzky-Golay (SG) filter was applied with third polynomial order and nine smoothing point. For the type of background subtraction, an automatic polynomial function was fitted to the spectrum and subtracted. Furthermore, the AUC extracted in this study is the integrated area under a specified peak of the spectrum and could be calculated using trapezoidal method on WITec Project Software or MatLab software.

#### 2.9.2. Principle Component Analysis

Multivariate data analysis was also performed on the WITec Project Software. For the study, Principle component analysis (PCA) was employed to further analysis the grouped spectra and reduce minor variations. PCA is the underlying method for many multivariate methods and could effectively obtain a reduced data set from multiple dimensions. Among the grouped Raman spectra, the first three principle components (PCs) are selected for reconstruction since they have contained most of essential information. In this study, PCA was applied for lipid conformation analysis. 

#### 2.9.3. Statistical Analysis

Spectra data were obtained from repeated measurements (*n* ≥ 18). The graphs were shown with mean values ± standard deviations (mean ± SD). Statistical differences were determined using one-way or two-way analysis of variance (ANOVA) followed by Student-Newman-Keuls (SNK) which were employed by GraphPad Prism 7.0 (GraphPad Software Inc., La Jolla, CA, USA). Diagrams and statistical differences were ultimately generated. Significant differences were marked with different number of asterisks: * *p* < 0.05, ** *p* < 0.01, *** *p* < 0.001.

## 3. Results

### 3.1. Lipid Content Analysis with Normalized Lipid Signal

**Figure 3:**
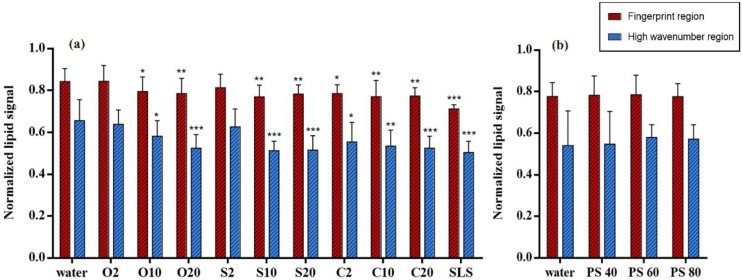
Normalized lipid signals in fingerprint and HWN region for lipid composition analysis of (**a**) PEG alkyl ethers treated SC and (**b**) PEG sorbitan esters treated SC. Mean ± SD, *n* = 18. * *p* < 0.05; ** *p* < 0.01; *** *p* < 0.001.

Lipid content in SC was analysed in the fingerprint and HWN regions by using lipid signals normalized by keratin signals. With the aim of detecting their impacts on lipids, different non-ionic emulsifiers were used to treat the SC, respectively. The alterations of lipid content are shown in Figure 3a,b. As can be seen, the red bars indicate the relative lipid content in fingerprint region while the blue bars represent the content in HWN region. It is evident that most of the PEG ethers cause a reduction of lipid content (Figure 3a) while all the polysorbate emulsifiers show no effects on SC lipid content (Figure 3b). Specifically speaking, the group of PEG ethers treated SC shows different extent of lipid reduction. Among them, only O2 and S2 treated SCs indicate no effects on SC lipid content. Focusing on the rest of the emulsifiers, all the PEG-20 alkyl ethers display to dramatically reduce lipid content. Regarding the PEG-10 alkyl ethers, O10 shows a relatively smaller difference compared to S10 and C10 which both indicate a greater reduction of skin lipid content. Interestingly, only C2 in PEG-2 alkyl ethers shows a slight impact on reducing lipid content. In contrast, polysorbate emulsifiers reflect completely no extraction of lipids. It turns out a part of this outcome is correlated with a previous result in our group that showed 5% of polysorbate 60 had no effects on lipids [13]. In general, the result of lipid content analysis in fingerprint region is complementary to that in HWN region and exhibited the same tendency of emulsifier effects.

### 3.2. CH_2_ Twisting and Scissoring Mode Analysis

**Figure 4:**
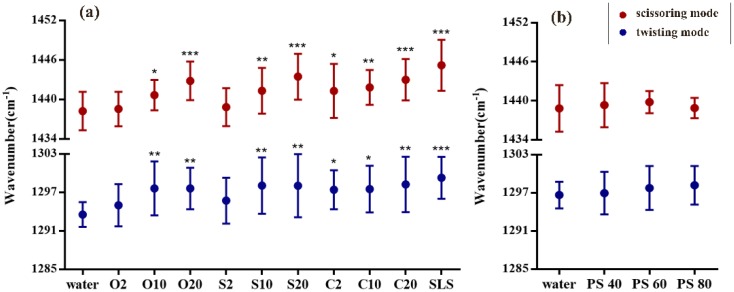
Lipid signals of CH_2_ twisting and scissoring mode for analyzing (**a**) PEG alkyl ethers treated SC and (**b**) PEG sorbitan esters treated SC. Mean ± SD, *n* = 18. * *p* < 0.05; ** *p* < 0.01; *** *p* < 0.001.

CH_2_ twisting mode in fingerprint region was selected in this study. It is also feasible to analyse the lipid conformational order. The band derived from CH_2_ twisting mode is located at about 1300 cm^−1^. The band shift is sensible to the conformation of hydrocarbon chains of lipids, so that it could be used as another conformational signal. Figure 4 shows the comparison of twisting mode between emulsifier treated and water treated skin samples. The peak location is in between 1285–1303 cm^−1^. It can be clearly seen that SLS has the most significant effect on SC lipid as the band shifts from about 1293 cm^−1^ to about 1299 cm^−1^. Among PEG ethers, relatively higher effects on lipids are caused by the PEG ether emulsifiers with the average number of oxyethylene groups of 20 as well as O10 and S10. Interestingly, C10 only shows small effects on lipid conformation. Nevertheless, in lipid content and C–C skeleton conformation analysis presented above, C10 has been determined to strikingly extract lipids and change conformational order. Apart from this slight discordance, the result is still a confirmation of emulsifier effects on lipid signals. Further, no significant effects are noted in polysorbate emulsifier treated SCs.

Except for the detailed information of hydrocarbon chains of lipids, CRS is also available to determine the lateral packing of lipids with CH_2_ scissoring mode in fingerprint region. Using the peak at 1434–1452 cm^−1^, Figure 4 shows the differences of scissoring mode when comparing emulsifier treated and water treated skin samples. It can be observed that some bands strikingly shift to higher wavenumbers after treatment with emulsifiers such as PEG-20 alkyl ethers and SLS. It means that the lateral packing tends to be transformed from orthorhombic phase to hexagonal or liquid-like phase. C10 and S10 shows to moderately lower the lateral packing density. However, the influence of O10 on SC is slightly lower than what we expected but similar to the impact of C10 on SC. In line with results from C–C skeleton vibration mode analysis, the results from scissoring mode analysis show no significant difference for polysorbate emulsifiers treated SCs. 

### 3.3. C–C Skeleton Conformation Analysis

**Figure 5:**
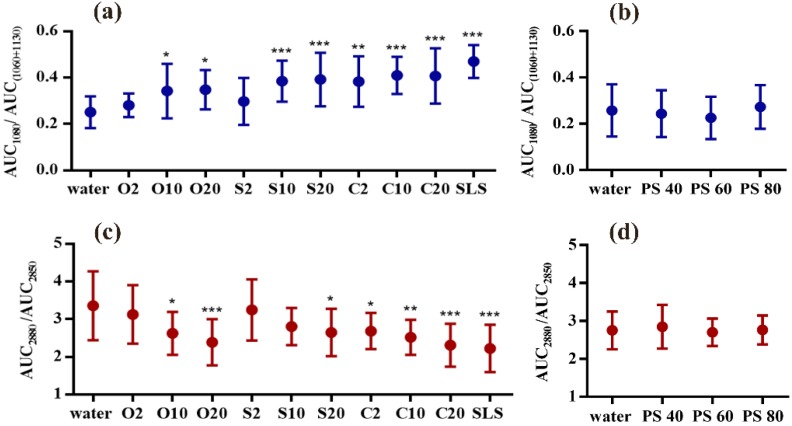
C–C skeleton vibration mode for lipid conformation analysis of (**a**) PEG alkyl ethers treated SC and (**b**) PEG sorbitan esters treated SC; lateral packing order analysis by AUC ratio of band 2850 cm^−1^ and 2880 cm^−1^: (**c**) PEG alkyl ethers treated SC and (**d**) PEG sorbitan esters treated SC. Mean ± SD, *n* = 18. * *p* < 0.05; ** *p* < 0.01; *** *p* < 0.001.

C–C skeleton vibrations in fingerprint region contain the all-*trans* signal (1060 cm^−1^ and 1130 cm^−1^) and *gauche* signal (1080 cm^−1^) of SC lipids. With the application of non-ionic emulsifiers, lipid conformation in SC was influenced to different degree. As displayed in Figure 5a, most of the PEG ethers show significant effects on lipid conformation when compared with water treated SC. Especially S10 and S20 as well as C10 and C20 present huge effects on SC lipid conformation, indicating the intercellular lipids have more gauche conformation (more disorder state). Surprisingly, O10 and O20 only show small influence on altering lipid conformation which is even less than the impact of C2. Whereas O2 and S2 show no effects on turning lipids into a more *gauche* conformation. Focusing on the investigation of the polysorbate group, it can be seen from Figure 5b that no significant difference has been found in polysorbate emulsifiers-treated SCs. It turns out that, with the application of polysorbate emulsifiers, lipid conformation remains in a more *trans* and ordered state.

### 3.4. Lateral Packing Analysis in HWN Region

Commonly, the broad shaped lipid-keratin peak originated from C–H vibration in HWN region is used to calculate lipid content and lipid lateral packing. The ratio of AUCs of 2880/2850 cm^−1^ has been defined to evaluate the lateral packing density of intercellular lipids. In this spectral region, peaks reflecting vibrations of SC lipids overlap with peaks derived from keratin. After the deconvolution process of lipids-keratin signals, the calculation of lateral packing in HWN is more precise owing to the exclusion of keratin peak influences. 

As the results shown in Figure 5c, O20 and C20 have the highest effects on decreasing the lateral packing density which are nearly the same as the effect of SLS. C10 tends to transform the lipid structure to hexagonal phase (more disordered structure). In addition, O10, S20 as well as C2 treated SC only indicate relatively lower effects. Whereas S10 treated SC unexpectedly presents no statistical difference which is unlike the lateral packing result shown in the scissoring mode analysis. The results depicted in Figure 5d reconfirmed that polysorbate emulsifiers appeared to exert no significant effects on SC lipids and maintained the orthorhombic structure of intercellular lipids. 

### 3.5. Skin Thickness Measurement

**Figure 6:**
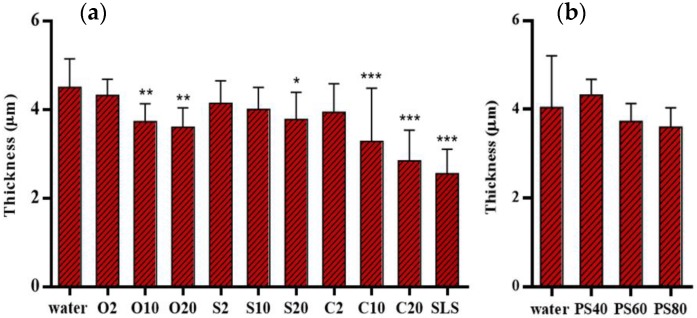
Thickness of SC after treatment with (**a**) PEG alkyl ethers treated SC and (**b**) PEG sorbitan esters treated SC. Mean ± SD, *n* = 18. * *p* < 0.05; ** *p* < 0.01; *** *p* < 0.001.

The thickness of skin samples was measured after the treatment with different emulsifiers. It has to be noted that SC thickness was measured on dried SC sheets and will thus give lower values as in (hydrated) full thickness skins. With the comparison to references, the results are shown in Figure 6. It is apparent from Figure 6a that the water treated SC was measured to be the thickest with the average thickness of 4.50 ± 0.64 μm. In contrast, SLS treated SC exhibited the thinnest skin samples (2.55 ± 0.54 μm), followed by the C20 and C10 treated SC with thickness of 2.84 ± 0.69 μm and 3.27 ± 1.20 μm respectively. O10 and O20 treated SC showed a tendency towards a reduced thickness as well. Unexpectedly, no indication was found of reduction of SC thickness after treatment with S10. In another experiment depicted in Figure 6b, the water treated SC was measured to have the thickness of 4.04 ± 1.16 μm, while the polysorbate emulsifiers treated SCs showed no significant difference when compared with water treated SC.

## 4. Discussion

Throughout this study, different spectral signals were utilized to analyse the effects of non-ionic emulsifiers on SC lipids. The result of lipid content analysis was achieved with the combination of lipid spectral signals from fingerprint region and HWN region. We could clearly see that PEG ethers were more capable to extract the lipids from SC than polysorbate emulsifiers. Meanwhile, both results of lipid content analysis were in good correlation with each other. It proved that after curve fitting process, the lipid-keratin peak ratio could serve as a sensitive lipid signal to calculate the lipid content. As an alternative, the lipid-keratin peak ratio in fingerprint region may be used and gives similar results.

The conformational analysis was evaluated in this study with the band shift of the twisting mode and calculated by the ratio of three small peaks related to *gauche/trans* conformation which originated from the C–C skeleton vibration bands. In view of the same tendency of detected emulsifier effects, both of the lipid spectral signals are capable to analyse lipid conformation accurately. However, comparing a series of complicated data processing steps in C–C skeleton vibration features, the prominent peak shift related to CH_2_ twisting mode at about 1300 cm^−1^ would be a better choice and less time-consuming.

There was a slightly difference in lipid analysis of lateral packing density. The ratio calculated by 2880/2850 cm^−1^ revealed that S10 had no significant effect. Whereas the results of scissoring mode indicated significant difference (*p* < 0.01). Although the deconvolution process has eliminated the influence of the keratin signal in the HWN region, the differences between results from S10 treated SC and water treated SC are not large enough to reach statistical significance. With the consideration of minor errors may result from the curve fitting process, the signal of scissoring mode might provide a more sensitive and efficient detection in our further study.

As the preliminary study of our group presented, the thinning of SC was assumptively caused by the extraction of lipids, subsequently leading to a loosened cohesion of SC and thereby fascinating keratinocytes removal [13]. In the skin thickness analysis of present work, most results complied with expectations and previous findings although the results of S10 and C2 reflected inconsistent with former lipid signals which may be triggered by the influence of different donors. The thinner the SC thickness originally, the less accurate the measuring result. Besides, the isolation of SC may also induce measurement errors.

Since this systematic study has proved that non-ionic emulsifiers have the potential to interact with SC lipids, we may find some rules or mechanisms to explain the ability of them to extract lipid components and decrease lipid order of SC. First, their capability to affect SC lipids might be governed by their structural properties as they contain both polar head region of hydrophilic chain and nonpolar tail region of hydrophobic chain. The characteristics of non-ionic emulsifiers used in this study has been listed in Table 1. As the influence of PEG ethers on SC shown, we may suggest that the higher the average number of oxyethylene groups, the stronger the interaction between PEG ethers and SC lipids. Meanwhile, the alkyl chain was highlighted in the potency of emulsifier interaction with SC lipid as well. With the results shown in this study, C2 appeared to be the only emulsifier to reduce lipid content and increase lipid disorder compared to other PEG-2 ethers. As they own the same number of oxyethylene groups, we may speculate that PEG derivatives with less carbon numbers of alkyl chain present higher ability to disturb SC lipid properties. Furthermore, keeping constant the number of carbon atoms in hydrophobic chain, similar effects of PEG oleyl ethers (unsaturated alkyl chain, C18) and PEG stearyl ethers (saturated alkyl chain, C18) on SC lipids were observed. We might then assume that with the same length of alkyl chain, the double bound only show little impact on SC lipids.

In the present study of polysorbate emulsifiers, they have been confirmed to have no influence on SC lipids, although they have longer polyoxyethylene chain (*n* = 20) and different length of alkyl chain. In this case, the molecular weight and structures could also serve as underlying factors for explaining the effects of polysorbate emulsifiers. The molecular structures of PEG ethers and esters used in this study are given in Table 2. It is apparent that polysorbate emulsifiers have larger molecular structure sizes which are expected to make it difficult for them to penetrate the skin and interact with SC lipids. Furthermore, although a recent finding from another group revealed a slight toxicity of polysorbate emulsifiers towards skin cells, it is not contradictory to our results that presented more friendly effects of them on skin lipids [49]. Our investigation suggests that polysorbates do not penetrate the skin and thus do not reach the deeper levels of the skin where living cells are located. Thus, it can be concluded that they may be regarded as safe excipients as long as they are not taken up into viable cell layers. Meanwhile, the emulsifier incorporated in various formulations may also display different influence on skin properties [13,49].

Last, according to the HLB values listed in Table 1, we first expect that the HLB values of non-ionic emulsifiers correlate to their effects on SC lipids. We could suggest from the result of PEG ethers that the higher the HLB value, the more intensive effects of emulsifiers on SC lipids. However, C2 with HLB value of 5.3 sometimes revealed relatively similar influence with O10 and S10 (HLB value 12.4). More strikingly is that all polysorbate emulsifiers present no effects on SC lipids with HLB values all around 15. Therefore, we may conclude that HLB value alone is not a reliable predictor of interactions between non-ionic emulsifiers and SC lipids. On the other hand, the molecular weight and structures may be the main factor of polysorbate emulsifiers to interact with skin lipids. Concerning these debatable factors, further research needs to be done for deeper understandings of possible mechanisms to describe how non-ionic emulsifiers penetrate the skin and interact with skin components.

## 5. Conclusions

This study systematically examined the effects of non-ionic emulsifiers, including PEG alkyl ethers and PEG sorbitan fatty acid esters, on SC lipids. CRS was employed in this study as a non-invasive, efficient and versatile instrument. Different spectral signals of CRS in both fingerprint and HWN regions were applied to assess the alteration of lipid content, conformation, lateral packing order and SC thickness which caused by the interaction of non-ionic emulsifiers with SC. To sum up, it has been demonstrated that the results of conformation and lateral packing order analysis were basically correlated with the results of content analysis, indicating that the extraction of lipids from SC may disturb the lipid structures in SC, and to some extent weaken the skin structural integrity. Furthermore, our results so far implied that non-ionic emulsifiers of polysorbates as well as PEG ethers with a smaller number of oxyethylene groups would be better choices to incorporate into topical formulations due to their lower effects regarding extraction of lipids, interruption of lipid organizations and further damage of skin barrier functions.

Overall, based on these findings, the assessment of different lipid signals played a meaningful role to filtrate more effective spectral features for deeper differentiations. This research on non-ionic emulsifiers could also serve as a basis and be helpful to screen suitable emulsifiers for further formulation development.

## Figures and Tables

**Table 1:** Characteristics of non-ionic emulsifiers including polyethylene glycol (PEG) alkyl ethers and PEG sorbitan esters used in this study.

Non-Ionic Emulsifiers	Alkyl Chain	Alkyl Chain LENGTH and Saturation	Number of Oxyethylene Group	Abbreviations	HLB Value
PEG-2 oleyl ether	Oleyl alcohol	C18, C9–C10 unsaturated	2	O2	5.0
PEG-10 oleyl ether	Oleyl alcohol	C18, C9–C10 unsaturated	10	O10	12.4
PEG-20 oleyl ether	Oleyl alcohol	C18, C9–C10 unsaturated	20	O20	15.3
PEG-2 stearyl ether	Stearyl alcohol	C18	2	S2	4.9
PEG-10 stearyl ether	Stearyl alcohol	C18	10	S10	12.4
PEG-20 stearyl ether	Stearyl alcohol	C18	20	S20	15.3
PEG-2 cetyl ether	Cetyl alcohol	C16	2	C2	5.3
PEG-10 cetyl ether	Cetyl alcohol	C16	10	C10	12.9
PEG-20 cetyl ether	Cetyl alcohol	C16	20	C20	15.7
PEG-20 sorbitan monopalmitate	Palmitic acid	C16	20	PS40	15.6
PEG-20 sorbitan monostearate	Stearic acid	C18	20	PS60	14.9
PEG-20 sorbitan monooleate	Oleic acid	C18, C9–C10 unsaturated	20	PS80	15

**Table 2:** Chemical structures of PEG alkyl ethers and PEG sorbitan fatty acid esters.

PEG Alkyl Ethers	Chemical Structures	PEG Sorbitan Fatty Acid Esters	Chemical Structures
PEG-n oleyl ether	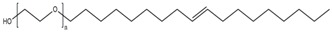	PEG-20 sorbitan monopalmitate (Polysorbate 40)	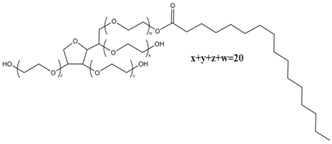
PEG-n stearyl ether	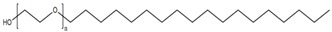	PEG-20 sorbitan monostearate (Polysorbate 60)	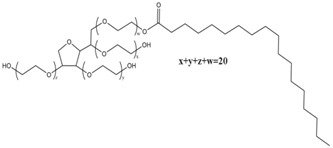
PEG-n cetyl ether	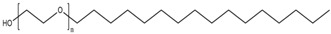	PEG-20 sorbitan monooleate (Polysorbate 80)